# Psychotomimetic symptoms after a moderate dose of a synthetic cannabinoid (JWH-018): implications for psychosis

**DOI:** 10.1007/s00213-021-05768-0

**Published:** 2021-01-26

**Authors:** Eef L. Theunissen, Johannes T. Reckweg, Nadia R. P. W. Hutten, Kim P. C. Kuypers, Stefan W. Toennes, Merja A. Neukamm, Sebastian Halter, Johannes G. Ramaekers

**Affiliations:** 1 Department of Neuropsychology and Psychopharmacology, Faculty of Psychology and Neuroscience, Maastricht University, P.O. Box 616, 6200 MD Maastricht, The Netherlands; 2 Department of Forensic Toxicology, Institute of Legal Medicine, Goethe University of Frankfurt, Frankfurt, Germany; 3 Institute of Forensic Medicine, Forensic Toxicology, Medical Center, Faculty of Medicine, University of Freiburg, Freiburg, Germany; 4 Hermann Staudinger Graduate School, University of Freiburg, Freiburg, Germany

**Keywords:** CB1 receptor agonists, Cannabimimetic, Spice, Schizophrenia, Clinical characteristics, Clinical study

## Abstract

**Background:**

Synthetic cannabinoids (SCs) are the largest class of novel psychoactive substances (NPS) and are associated with an increased risk of overdosing and adverse events such as psychosis. JWH-018 is one of the earliest SCs and still widely available in large parts of the world. Controlled studies to assess the safety and behavioural profiles of SCs are extremely scarce.

**Aim:**

The current study was designed to assess the psychotomimetic effects of a moderate dose of JWH-018.

**Methods:**

Twenty-four healthy participants (10 males, 14 females) entered a placebo-controlled, double blind, within-subjects trial and inhaled vapour of placebo or 75μg/kg bodyweight JWH-018. To ascertain a minimum level of intoxication, a booster dose of JWH-018 was administered on an as-needed basis. The average dose of JWH-018 administered was 5.52 mg. Subjective high, dissociative states (CADSS), psychedelic symptoms (Bowdle), mood (POMS) and cannabis reinforcement (SCRQ) were assessed within a 4.5-h time window after drug administration.

**Results:**

JWH-018 caused psychedelic effects, such as altered internal and external perception, and dissociative effects, such as amnesia, derealisation and depersonalisation and induced feelings of confusion.

**Conclusion:**

Overall, these findings suggest that a moderate dose of JWH-018 induces pronounced psychotomimetic symptoms in healthy participants with no history of mental illness, which confirms that SCs pose a serious risk for public health.

## Introduction

Smoking mixtures containing synthetic cannabinoids (SCs) are being sold as alternatives for cannabis since about 2008. Typical brand names of smoking mixtures include Spice, K2 and Yucatan Fire, but hundreds of brands have flooded the market. These mixtures do not contain cannabis but are smoked to experience a cannabis-like high. Originally, these blends were portrayed as natural and harmless products and were easily accessible, which made them a very popular replacement for cannabis, especially in countries where natural cannabis is illegal or less easily accessible (European Monitoring Centre for Drugs and Drug Addiction 2019, 2017). It soon became clear, however, that SCs are commonly associated with adverse events such as tachycardia, agitation and nausea, while severe adverse events (e.g. stroke, seizure and psychosis) and associated deaths are less common (Tait et al. 2016; Darke et al. 2020).

Since their introduction on the market, many countries have banned an increasing number of SCs. Yet, manufacturers have been able to bypass these regulations by modifying the chemical structure and synthesise new SCs that are not covered by legislation and are not detectable by standard drug screens (De Luca and Fattore 2018). As a consequence, SCs have become a large and fast growing class of new psychoactive substances (NPS) on the market (United Nations Publications 2019). By 2018, about 190 SCs have been reported to the EMCDDA (European Monitoring Centre for Drugs and Drug Addiction 2019). While the number of newly emerging SCs has slowed during the last couple of years, they still make up for 30% of the NPS reported to United Nations Office on Drugs and Crime, indicating that they are still widely used (United Nations Office on Drugs and Crime (UNODC) 2020).

Similar to Δ9-tetrahydrocannabinol (THC), the active principal component of cannabis, SCs bind to the central cannabinoid receptors (CB1 and CB2). Compared with THC, the majority of SCs have a much higher binding affinity for CB1 receptors, and they often act as full agonists (Uttl et al. 2018; Gurney et al. 2014; Castaneto et al. 2014). Therefore, the effects of SCs on psychological state and physiological functioning can be much stronger than that of natural cannabis, and the risk for overdosing is considerably higher particularly in inexperienced users. This is especially problematic with the newest generation of SCs (De Luca and Fattore 2018), which are up to 85 times as potent as THC (Adams et al. 2017). These SCs can cause severe adverse events and have been related to numerous hospital admissions and even deaths (Hermanns-Clausen et al. 2018; World Health Organization 2016; Darke et al. 2020). In addition, abuse liability of SCs has been demonstrated in animal studies (Gatch and Forster 2019; Tai and Fantegrossi 2014; Zanda and Fattore 2018) and is suggested to be even greater than that of cannabis (Fantegrossi et al. 2014).

The effects of natural cannabis on physiological, behavioural and psychological outcome measures are well known and have been investigated in experimental, placebo-controlled studies. In recreational users, THC in doses between 40 and 500 μg THC/kg body weight generally causes elevated heart rate, decreased blood pressure and impairment of cognitive and psychomotor functions (Ranganathan and D'Souza 2006; Bosker et al. 2012; Curran et al. 2002; Hall and Solowij 1998; Lundqvist 2005; Lichtman et al. 2002; Ramaekers et al. 2006; Hall and Degenhardt 2009). In addition, THC can produce psychedelic effects such as altered perception of the body, environment and time (Zuurman et al. 2008; Van Wel et al. 2015), as well as psychosis-like (psychotomimetic) symptoms (e.g. positive and negative symptoms, perceptual alterations, euphoria and anxiety) during intoxication (D'Souza et al. 2004). Dissociation, which is ‘a disruption of and/or discontinuity in the normal integration of consciousness, memory, identity, emotion, perception, body representation, motor control and behaviour’ (American Psychiatric Association 2013), is shown to be an important factor underlying vulnerability to psychotic experiences (Longden et al. 2020). Dissociative symptoms have been reported during cannabis intoxication and are known to also occur in psychiatric disorders such as schizophrenia (Hunter et al. 2004). Cannabis-induced dissociative symptoms have been reported even to exceed those observed in schizophrenia patients (van Heugten-Van der Kloet et al. 2015).

Case studies and epidemiological data affirm the link between the use of cannabis and psychosis (Every-Palmer 2010; Papanti et al. 2013; Peglow et al. 2012; Fattore 2016). In individuals with a preexisting psychotic disorder, cannabis can aggravate psychotic symptoms or induce a relapse, while it can evoke transient psychotomimetic symptoms in people with no prior history of the disorder (Volkow et al. 2016; Radhakrishnan et al. 2014). Importantly, the risk of an adverse psychotomimetic experience is dose related and increases with higher doses of cannabis (Radhakrishnan et al. 2014; D'Souza et al. 2004; Di Forti et al. 2015). This is particularly problematic in the context of highly potent SCs that can easily be overdosed because their specific potencies are most often unknown and come unexpected to users. Likewise, safety and toxicology data on SCs is missing because controlled studies with these compounds are virtually absent.

JWH-018 was the first SC identified in Spice in 2008 (Steup n.d.). It is a widely known synthetic cannabinoid and has been widespread for years (World Health Organization 2014). It was scheduled in several countries, including the USA, in 2011, and subsequently replaced by new, more potent, compounds (Seely et al. 2012; United Nations office on Drugs and Crime 2013). Many of these compounds are structurally similar to JWH-018 (Musah et al. 2012; Alam and Keating 2020; World Health Organization 2016). The use of JWH-018 however never vanished entirely, and it is still prevalent in large parts of the world (Oberenko et al. 2019; Vučinić et al. 2018; Darke et al. 2020).

To date, JWH-018 is the only SC that has been studied in controlled clinical trials. So far, these typically concerned small-scaled efforts designed to determine the safety profile and the minimum to moderate effective dose of this compound (Theunissen et al. 2019; Theunissen et al. 2018). These efforts have paved the way for the present up-scaled study in healthy volunteers that was designed to assess the psychotomimetic effects of a moderate dose of JWH-018 and to evaluate the implication for psychosis.

## Materials and methods

The study was approved by the standing Medical Ethics Committee of Maastricht University and was carried out in compliance with the current revision of the Declaration of Helsinki (amended in 2013, Fortaleza) and the International Conference on Harmonization guidelines for Good Clinical Practice. A permit for obtaining, storing and administering JWH-018 was obtained from the Dutch drug enforcement administration. All participants gave written informed consent and received financial compensation for their participation.

### Participants

A total of 24 occasional users of cannabis were included in the study. This sample size is comparable with previous studies investigating the dissociative effects of cannabis (van Heugten-Van der Kloet et al. 2015). Participants recruited via advertisements were screened using a locally developed health questionnaire and underwent a medical examination (including an electrocardiogram (ECG), haematology and blood chemistry, urinalysis and drug and pregnancy screening). The following inclusion criteria applied: occasional use of cannabis (participants had minimum 1 year experience with cannabis, with a minimum and maximum use of 12 and 120 times/year), free from psychotropic medication; good physical health as determined by medical examination and laboratory analysis (haematology, blood chemistry and urinalysis); absence of any major medical, endocrine and neurological condition; body mass index (weight/length^2^) between 18 and 28 kg/m^2^; written informed consent. Exclusion criteria were excessive drinking (> 20 alcoholic consumptions/week), pregnancy or lactation or failure to use contraceptives, hypertension (diastolic > 90 mmHg; systolic > 140 mmHg), history of psychiatric disorders and history of drug abuse.

### Design and treatments

The study was conducted according to a placebo-controlled, double-blind, within-subjects design. On separate test days, each participant inhaled the vapour of a placebo or a minimum dose of 75μg/kg bodyweight JWH-018. The order of treatments was counterbalanced, with half of the participants receiving placebo first, while the other half received JWH-018 first. Test days were separated by a minimum washout period of 7 days to avoid cross-condition contamination.

JWH-018 powder was purchased from THC-pharm (Germany). Knaster Hemp (Zentauri, Germany), a herbal blend with hemp aroma (0% THC), was used as placebo. Both were heated via a vaporiser pen (Puffco® plus) reaching approximately 380°C, high enough for JWH-018 to evaporate. Participants inhaled the vapour in five intakes, according to a strict inhalation regimen (i.e. inhaling for 5 s, holding breath for 5 s, followed by exhaling). In case participants did not show a subjective response (i.e. if their subjective high score was < 30% of the maximum possible response, see ‘Subjective high’) within 15 min after administration of JWH-018, a booster dose of 50 μg/kg bodyweight was administered. A researcher who was not involved in the study assessments was responsible for the vaporiser preparation, administration and checking the subjective high score. Participants who were given a booster dose were not told that this was due to the low score on the subjective high scale but were made to believe that something went wrong during administration. In order to keep the researcher blind, this was also performed a couple of times after placebo administration.

On average, participants received 4.97 mg (min = 3.75; max = 6.67 mg) of JWH-018 during the first administration. Four participants did not show a subjective response (i.e. their subjective score was below 30% of the maximum score) within 15 min after administration and were therefore given a booster dose (average = 3.26 mg). The total average dose was 5.52 mg.

### Subjective questionnaires

#### Subjective high

Subjective high is self-rated on a 10-cm visual analogue scale (VAS), with 0 indicating ‘not high at all’ and 10 indicating ‘extremely high’. Subjective high was rated at regular intervals during the test day.

#### CADSS

The Clinician-Administered Dissociative States Scale (CADSS) (Bremner et al. 1998) comprises 19 self-rated items, ranging from 0 ‘not at all’ to 4 ‘extremely’. It is divided into three components: *depersonalisation* (5 items), *derealisation* (12 items) and *amnesia* (2 items). A total dissociative score is achieved by summing all items. The CADSS is designed to be a standardised measure of present-state dissociative symptomatology and was previously found to be sensitive to dissociative effects of psychedelics and drugs of abuse, such as ketamine and THC (Derntl et al. 2019; van Heugten-Van der Kloet et al. 2015; D'Souza et al. 2004). The CADSS was administered at 5 min and 4 h after drug intake or booster dose.

#### Bowdle visual analogue scales (Bowdle)

Psychedelic symptoms are assessed using a 13-item VAS (Bowdle et al. 1998). Two scales measure subjective ‘high’ and ‘drowsiness’. From the other scales, composite scores of ‘*internal perception*’ (reflecting inner feelings that do not correspond with reality) and ‘*external perception*’ (reflecting a misperception of an external stimulus or a change in the awareness of the subject's surroundings) are calculated (Zuurman et al. 2008). The Bowdle was administered 1 h after drug intake or booster dose.

#### POMS

The Profile of Moods States (POMS) is a self-assessment mood questionnaire with 72 items, rated on a 5-point Likert scale, with 0 being not at all to 4 extremely. Participants have to indicate to what extent these items were representative of their mood at that moment in time. Eight mood states are classified and quantified by calculating the sum score of associated items for each mood state, i.e. *anxiety*, *depression*, *anger*, *vigour*, *fatigue*, *confusion*, *friendliness* and *elation*. Two composite scales are derived, *arousal* and *positive* mood (de Wit et al. 2002). The POMS was administered at baseline and at 1 h after drug intake or booster dose.

#### Sensitivity to Cannabis Reinforcement Questionnaire (SCRQ)

This questionnaire asks participants to rate their liking and wanting of cannabis use during their present condition and in general. Participants are asked four questions: How pleasant is using cannabis right now (drug liking)? How much do you want to use cannabis right now (drug wanting)? How pleasant is using cannabis in general? How much do want to use cannabis in general? Subjective valence of liking and wanting is scored on a 5-points scale. The SCRQ was administered about 5 min after drug intake or booster dose.

### Safety and pharmacokinetics

Safety measurements (heart rate and blood pressure) and blood samples for pharmacokinetic assessments were taken at regular intervals after administration (For a detailed description, see Theunissen et al. 2021).

### Procedures

Participants were asked to refrain from using alcohol or caffeine on the test day and the day prior to testing. Smoking was prohibited for 30 min before and during test days. Participants were asked to arrive at the testing facilities well rested. On each test day, participants were instructed to have a standard breakfast before coming to the site, while they received lunch at the site. Participants were instructed to continue their cannabis use as normal but were requested to abstain from cannabis from about 5 days before the test day to make sure they had a negative urine drug screen on the test day.

Test days took place at the testing facilities at Maastricht University. An alcohol breath test and a urine drug screen to assess the presence of alcohol, morphine, cocaine, cannabis, methamphetamine or amphetamine were performed upon arrival. A urine pregnancy test was also performed in women. When all tests turned back negative, administration was performed, and within the next 4.5 h, a battery of subjective questionnaires and cognitive tests (described in (Theunissen et al. 2021)) was performed together with safety measurements and blood sampling. See Table 1 for an overview of the timings of the subjective questionnaires.

**Table 1:** Overview of the time of the subjective questionnaires taken during the test day relative to drug administration

Time*	Subjective high	POMS	CADSS	Bowdle	SCRQ
baseline	x	x			
5 min	x		x		x
15 min	x				
30 min	x				
45 min	x				
1 h	x	x		x	
1h30					
2 h	x				
2h30					
3 h	x				
4 h	x		x		

*Relative to time of administration or the last booster dose in cases where this was needed

Participants were discharged when they had a score lower than 1 on VAS scales measuring intoxication and sedation, and the experimenters judged that they were no longer intoxicated or sedated.

### Statistical analyses

Data were analysed using a GLM Univariate ANOVA with Drug (placebo and JWH-018) as a within-subject factor. In cases where a test was repeated more than once on a test day, a GLM repeated-measures ANOVA, with Drug (placebo and JWH-018) and Time as within-subject factors, was used. A Greenhouse-Geisser correction was applied in case of violation of sphericity. In the case of significant drug × time interactions, separate drug-placebo contrasts were conducted, and sequential Bonferroni correction was applied to correct for multiple comparisons. A *p* value of <.05 was considered statistically significant. All statistical tests were conducted using IBM SPSS statistics, version 26.

In the JWH-018 condition, data from one participant were missing for the POMS at 1h post-administration. These data were not replaced.

## Results

Data from 24 participants (10 males, 14 females) was analysed. On average (SD, min-max), participants were 22.8 years old (3.05, 18.9–33.6) and used cannabis for 4.5 years (2.15, 1–9) and this 3.4 times a month (2.3, 1–10).

### Safety and pharmacokinetics

After JWH-018 administration, three participants reported nausea and/or stomach ache, while three participants reported dry mouth. Two participants reported a short moment of increased energy followed by a feeling of tiredness, while a third participant only reported sedative feelings. Two participants reported headaches, and one participant reported paranoid feelings. In the placebo condition, five participants reported headaches, while one participant reported dizziness.

**Fig. 1:**
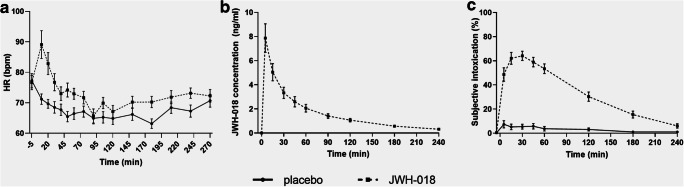
Mean (SEM) heart rate (HR) over time for both JWH-018 and placebo (**a**). Concentrations of JWH-018 in serum, (**b**). Subjective high score for JWH-018 and placebo (**c**)

Mean (SE) heart rate is shown in Fig. 1a. JWH-018 significantly increased heart rate, especially within the first hour after administration. Blood pressure, on the other hand, was not affected by the drug.

Mean (SE) concentrations of JWH-018 in serum are shown in Fig. 1b. Peak concentration was 8.00 ng/mL (SD = 2.81, min-max: 1.07–22.45) and was reached 5 min after administration.

### Subjective high

Five minutes after administration (or booster), the average subjective intoxication was 49% on VAS, while maximal subjective intoxication was reached 30 min post-administration (64% on VAS) (see Fig. 1c).

### Clinician-Administered Dissociative States Scale

**Fig. 2:**
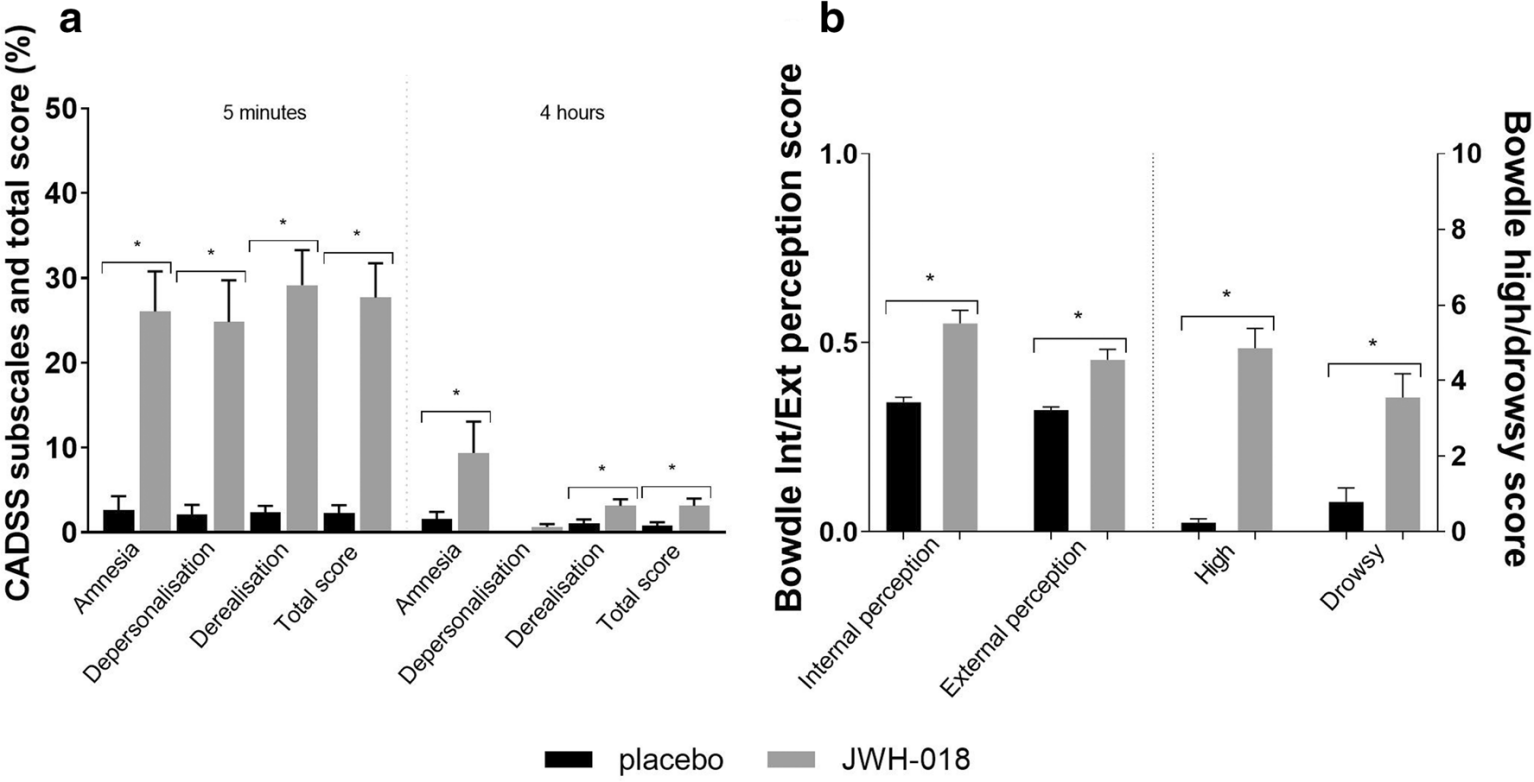
Mean (SE) scores (percentage of the maximum score) on the CADSS scales amnesia, derealisation and depersonalisation and total score at 5 min and 4 h after JWH-018 and placebo administration (**a**). Mean (SE) scores on the Bowdle scales internal (Int) and external perception (Ext) (left y-axis) and high and drowsiness (right y-axis) after JWH-018 and placebo administration, (**b**). *Significant drug-placebo contrast (sequential Bonferroni corrected)

A main effect of drug was found on amnesia (*F*_1,23_= 11.81; *p*= .002; η_p_^2^= 0.34), depersonalisation (*F*_1,23_= 26.95; *p* <.001; η_p_^2^= 0.54), derealisation (*F*_1,23_= 43.89; *p* <.001; *η*_*p*_^2^= 0.66), and total score (*F*_1,23_= 41.69; *p* < .001; *η*_*p*_^2^= 0.64). All scales also demonstrated a significant main effect of time and a drug × time interaction, demonstrating that the JWH-induced effect wore off over time (see Fig. 2a). Drug-placebo contrasts revealed that JWH-018 increased symptoms of amnesia (*p* < .001), derealisation (*p* < .001), dissociation (*p* < .001) and the total score (*p*< .001) at 5 min after administration. After 4 h, these drug-induced effects were less strong but still statistically significant for derealisation (*p* < .0125) and total score (*p* < .016).

### Bowdle

The Bowdle VAS scores for external and internal perception were significantly different between drug conditions (*F*_1,46_= 32.33; *p* < .001; *η*_*p*_^2^ = 0.41; *F*_1,46_= 19.71; *p* < .001; *η*_*p*_^2^= 0.30). This was also the case for the scales high and drowsiness (*F*_1,46_= 74.61; *p* < .001; *η*_*p*_^2^ = 0.62; *F*_1,46_= 14.84; *p* < .001; *η*_*p*_^2^ = 0.24). JWH-018 caused an increase on all scales as compared to placebo (Fig. 2b).

### Profile of Mood States

**Fig. 3:**
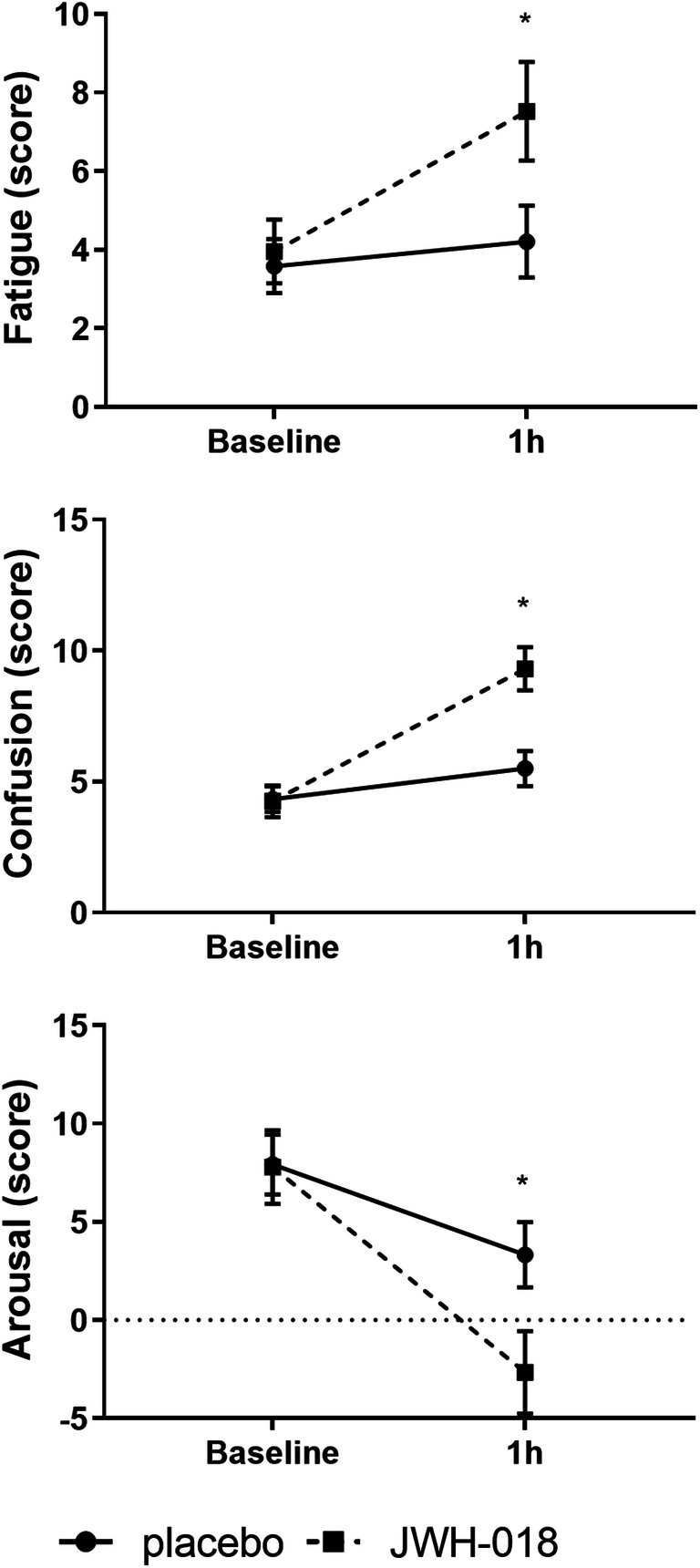
Mean (SE) scores on the POMS scales Fatigue, Confusion and Arousal at baseline and 1h after JWH-018 and placebo administration. *Significant drug-placebo contrast (*p*< .05)

The POMS scales Vigour, Fatigue, Confusion, Friendliness, Elation, Arousal and Positive mood all showed a significant main effect of Drug (*F*_1,22_ > 4.64; *p* < .042). For Fatigue, Confusion and Arousal, a significant drug × time interaction was also demonstrated (*F*_1,22_ > 4.49; *p* < .046) (see Table 2 for an overview of the test results, and Fig. 3 for the mean scores on the separate scales). At baseline, drug-placebo contrast did not show differences between drug conditions. One hour after drug administration, JWH-018 revealed increments in Fatigue (*p*= .004), Confusion (*p* < .001) and Arousal (*p* = .016), compared with placebo. No significant effects were found on the other mood states of the POMS, i.e. Depression, Anger or Anxiety.

**Table 2:** Overview of the GLM RM ANOVA test results for factor Drug, Time and Drug × Time of the POMS subscales

	Drug			Time			Drug × time		
POMS	*F*_1,22_	*p*	*η*_*p*_^2^	*F*_1,22_	*p*	*η*_*p*_^2^	*F*_1,22_	*P*	*η*_*p*_^2^
Vigour	23.72	<.001	0.52	0.23	NS	.01	0.00	NS	0.00
Fatigue	9.46	.006	0.30	7.11	.014	.24	7.57	.012	0.26
Confusion	40.67	<.001	0.65	12.85	.002	.37	22.02	<.001	0.50
Friendliness	4.64	.042	0.17	0.08	NS	.00	0.12	NS	0.00
Elation	14.51	.001	0.40	0.59	NS	.03	0.34	NS	0.02
Arousal	32.06	<.001	0.59	4.86	.038	.18	4.49	.046	0.17
Positive mood	8.12	.009	0.27	0.66	NS	.03	0.01	NS	0.00

### Sensitivity to Cannabis Reinforcement Questionnaire

**Fig. 4:**
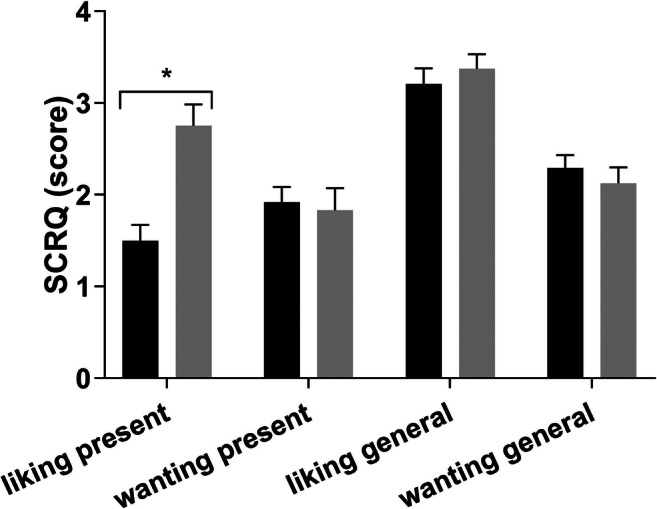
Mean (SE) scores on the SCRQ (5 min after JWH-018 and placebo administration). *Significant drug-placebo contrast (*p*< .05)

After JWH-018 administration, present drug liking increased (*F*_1,46_=18.55; *p* < .001; *η*_*p*_^2^= 0.29). The other items of the SCRQ did not differ between JWH-018 and placebo. See Fig. 4 for average (SE) scores.

## Discussion

The current study is the largest so far to assess the psychotomimetic effects of a SC in humans in a controlled manner. Twenty-four participants received a dose of at least 75 μg/kg bodyweight JWH-018 and placebo on separate occasions. On average, participants received a total dose of 5.52 mg of JWH-018. With the current administration procedure, we achieved adequate levels of subjective high comparable with those observed after single-dose administrations of cannabis (THC 14.5–33 mg) in similar experimental studies (Theunissen et al. 2012; Hartman et al. 2016). While peak serum concentration of JWH-018 (8.0 ng/mL) was similar to what we have demonstrated previously (7.49 ng/mL) (Theunissen et al. 2019), the current administration procedure led to more steady levels (SD = 2.81 vs. 5.66 ng/mL in our prior study). Blood concentrations of JWH-018 in intoxication cases (non-fatal as well as fatal) show a large variability (ranging from <.05 to 199 ng/ml) (Adamowicz 2020). However, these samples are taken hours or days after drug intake and are therefore an underestimation of the peak concentration of the drug. It is therefore fair to say that we used a moderate dose of JWH-018 in the current study.

One of the most frequently reported acute effects of SCs is an altered mental state, often described as the ‘zombie’ effect (Müller et al. 2010; Adams et al. 2017; Hermanns-Clausen et al. 2013a). This includes psychotomimetic and dissociative effects, which have been noted in up to 28% of the people who admitted the use of a SC (Vandrey et al. 2012; Forrester et al. 2012). In cases where toxicological analyses confirmed the presence of SCs, up to 38% of the users reported perceptual changes or hallucinations (Hermanns-Clausen et al. 2013b). We were able to confirm the presence of dissociative and psychedelic effects in our sample of healthy volunteers during JWH-018 intoxication. A moderate dose of JWH-018 caused psychedelic effects such as altered internal and external perception, and dissociative effects, such as amnesia, derealisation and depersonalisation and induced feelings of confusion. Overall, these findings suggest that a moderate dose of JWH-018 induces psychotomimetic effects in healthy participants with no history of mental illness. Acute intoxication by JWH-018, also led participants to score higher on drug compulsivity and drug liking. Compulsive craving is a typical feature of drug dependence (Heishman et al. 2001); hence, these effects demonstrate the abuse liability of JWH-018, similar to that shown previously for SCs in rodents (Gatch and Forster 2019; Zanda and Fattore 2018; Fantegrossi et al. 2014).

From natural cannabis, it is known that it can cause psychotomimetic symptoms in healthy volunteers and induce psychosis in vulnerable people (Johns 2001; Henquet et al. 2005). Cannabis strains that contain high levels of THC have been suggested to be more likely to produce psychotomimetic effects. In contrast, the simultaneous presence of high levels of CBD, a non-psychoactive constituent of cannabis, may protect the user for THC’s psychotic effect (Di Forti et al. 2009; Iseger and Bossong 2015). Psychotomimetic effects as observed after a moderate dose of JWH-018 have also been observed after single-dose administrations of cannabis containing up to 20 mg of THC (Van Wel et al. 2015; Zuurman et al. 2008; Solowij et al. 2019; van Heugten-Van der Kloet et al. 2015). The dissociative state resulting from JWH-018, however, seems to be more pronounced than that of THC. Drug-placebo difference on total dissociative score after inhaled THC (between 8-20 mg) (Van Wel et al. 2015; Solowij et al. 2019; van Heugten-Van der Kloet et al. 2015) and intravenous THC (5 mg) (D'Souza et al. 2004) ranged between 6.5 and 10.5, whereas in the current study, an increase of 19.25 was demonstrated. Although statistical evaluations are needed in order to draw reliable conclusions about a direct comparison between JWH-018 and THC, these data suggest that the dissociative effects following JWH-018 are more apparent.

Synthetic cannabinoids produce stronger and more frequent psychotic effects because they are potent and full CB1 agonists (Fattore 2016). THC, on the other hand, is a partial agonist. The currently studied SC, JWH-018, has an affinity for the CB1 receptor, which is five times greater than that of THC in natural cannabis (Aung et al. 2000). Therefore, it comes as no surprise that its psychotomimetic effects are stronger than those of natural cannabis. Psychotomimetic symptoms are especially concerning for people at risk for developing psychosis. Every-Palmer (2010), e.g. reported that consumption of smoking mixtures containing the SC CP47,497 and/or JWH-018 led to a re-emergence of psychosis (symptoms included agitation, delusions and disorganisation) in patients with a mental illness who were stable up until the use of the SC. In patients with serious mental illnesses, a JWH-018 containing smoking mixture, led to anxiety and psychotic symptoms in 69% of the users (Every-Palmer 2011). The fact that SCs are readily available, easily trafficked, cheap and not detected with standard drug tests makes them a serious concern for psychiatric hospitals and prisons (European Monitoring Centre for Drugs and Drug Addiction 2019).

CB1 receptors are widely distributed in brain areas implicated in the putative neural circuitry of psychosis, including the mesolimbic pathway (Mackie 2005; Herkenham et al. 1990; Goldstein et al. 1999; Buchsbaum 1990; Andreasen and Pierson 2008; Fornito et al. 2008). More research is however needed to fully understand how synthetic cannabinoids produce psychotomimetic effects or induce psychosis.

CB1 receptor antagonists, such as rimonabant and CBD, might be valuable candidates for reversing the acute psychotic effects of SCs (Meredith et al. 2020). Both drugs reduced activity in the mesolimbic cortex in animals (Alonso et al. 1999; Roser et al. 2010). In humans, CBD was also found to reverse THC-induced psychological effects (Schubart et al. 2014; Hallak et al. 2011). In animals, rimonabant has been shown to prevent the SC’s adverse effects such as ataxia and vomiting (Hruba and McMahon 2017) and produce an atypical antipsychotic effect (Ballmaier et al. 2007), though convincing evidence from human experimental studies is lacking (Meltzer et al. 2004; Kelly et al. 2011). Rimonabant’s adverse effects have led to the withdrawal of the drug. However, such adverse effects are not to be expected after single-dose administrations of a CB1 antagonist. Nonetheless, further research is needed to study whether CB1 antagonists or CBD could serve as antidotes for acute intoxication caused by SCs.

JWH-018 is one of the first SCs that appeared in smoking mixtures, but more potent SCs have taken over the market in more recent years. This implies that the risks of newer SCs to produce strong psychotomimetic effects are higher compared with JWH-018. This seems to be supported by some anecdotal case and hospital reports (Mörkl et al. 2016; Hermanns-Clausen et al. 2018). There is an urgent need for more controlled experimental studies to follow up on these developments in the drug market. However, getting ethical approval for these types of studies is challenging and time-consuming.

In the current study, we were able to demonstrate that healthy volunteers who are intoxicated by a moderate dose of the synthetic cannabinoid JWH-018 experience psychedelic and dissociative symptoms and feelings of confusion. These findings suggest that a moderate dose of JWH-018 induces pronounced psychotomimetic effects in healthy participants with no history of mental illness. It is speculated that psychotomimetic effects of recent and more potent SCs will even be more substantial, posing an even higher risk on public health.
